# Mesenchymal stem cells transplantation combined with IronQ attenuates ICH-induced inflammation response via Mincle/syk signaling pathway

**DOI:** 10.1186/s13287-023-03369-6

**Published:** 2023-05-15

**Authors:** Guoqiang Yang, Jiraporn Kantapan, Maryam Mazhar, Xue Bai, Yuanxia Zou, Honglian Wang, Bingfeng Huang, Sijing Yang, Nathupakorn Dechsupa, Li Wang

**Affiliations:** 1 Molecular Imaging and Therapy Research Unit, Department of Radiologic Technology, Faculty of Associated Medical Sciences, Chiang Mai University, Chiang Mai, Thailand; 2 Center of Radiation Research and Medical Imaging, Department of Radiologic Technology, Faculty of Associated Medical Sciences, Chiang Mai University, Chiang Mai, Thailand; 3 Research Center for Integrated Chinese and Western Medicine, The Affiliated Traditional Chinese Medicine Hospital of Southwest Medical University, Luzhou, China; 4 Acupuncture and Rehabilitation Department, The Affiliated Traditional Chinese Medicine Hospital of Southwest Medical University, Luzhou, China; 5 National Traditional Chinese Medicine Clinical Research Base and Drug Research Center of the Affiliated Traditional, Chinese Medicine Hospital of Southwest Medical University, Luzhou, China; 6 Institute of Integrated Chinese and Western Medicine, Southwest Medical University, Luzhou, China; 7 Department of Neurology and National Traditional Chinese Medicine Clinical Research Base, The Affiliated Traditional Chinese Medicine Hospital of Southwest Medical University, Luzhou, 646000 China; 8 Department of Magnetic Resonance Imaging, The Affiliated Traditional Chinese Medicine Hospital of Southwest Medical University, Luzhou, China

**Keywords:** Mesenchymal stem cells, IronQ, Intracerebral hemorrhage, Mincle, Inflammatory response

## Abstract

**Background:**

Intracerebral hemorrhage (ICH) is a severe brain-injured disease accompanied by cerebral edema, inflammation, and subsequent neurological deficits. Mesenchymal stem cells (MSCs) transplantation has been used as a neuroprotective therapy in nervous system diseases because of its anti-inflammatory effect. Nevertheless, the biological characteristics of transplanted MSCs, including the survival rate, viability, and effectiveness, are restricted because of the severe inflammatory response after ICH. Therefore, improving the survival and viability of MSCs will provide a hopeful therapeutic efficacy for ICH. Notably, the biomedical applications of coordination chemistry-mediated metal-quercetin complex have been verified positively and studied extensively, including growth-promoting and imaging probes. Previous studies have shown that the iron-quercetin complex (IronQ) possesses extraordinary dual capabilities with a stimulating agent for cell growth and an imaging probe by magnetic resonance imaging (MRI). Therefore, we hypothesized that IronQ could improve the survival and viability of MSCs, displaying the anti-inflammation function in the treatment of ICH while also labeling MSCs for their tracking by MRI. This study aimed to explore the effects of MSCs with IronQ in regulating inflammation and further clarify their potential mechanisms.

**Methods:**

C57BL/6 male mice were utilized in this research. A collagenase I-induced ICH mice model was established and randomly separated into the model group (Model), quercetin gavage group (Quercetin), MSCs transplantation group (MSCs), and MSCs transplantation combined with IronQ group (MSCs + IronQ) after 24 h. Then, the neurological deficits score, brain water content (BWC), and protein expression, such as TNF-α, IL-6, NeuN, MBP, as well as GFAP, were investigated. We further measured the protein expression of Mincle and its downstream targets. Furthermore, the lipopolysaccharide (LPS)-induced BV2 cells were utilized to investigate the neuroprotection of conditioned medium of MSCs co-cultured with IronQ in vitro.

**Results:**

We found that the combined treatment of MSCs with IronQ improved the inflammation-induced neurological deficits and BWC in vivo by inhibiting the Mincle/syk signaling pathway. Conditioned medium derived from MSCs co-cultured with IronQ decreased inflammation, Mincle, and its downstream targets in the LPS-induced BV2 cell line.

**Conclusions:**

These data suggested that the combined treatment exerts a collaborative effect in alleviating ICH-induced inflammatory response through the downregulation of the Mincle/syk signaling pathway following ICH, further improving the neurologic deficits and brain edema.

**Supplementary Information:**

The online version contains supplementary material available at 10.1186/s13287-023-03369-6.

## Introduction

Intracerebral hemorrhage (ICH), a severe stroke syndrome, possesses a disproportionate amount of stroke-induced neurological morbidity and mortality, which is estimated to have more than 5 million brain hemorrhage cases and nearly 2.8 million deaths worldwide each year [1–5]. Although less common than ischemic stroke (IS), ICH is the primary reason for stroke-induced mortality, and there is not yet a critical therapy beyond supportive care in clinical practice [6–9]. After ICH, severe intracranial hematoma permanently destroys normal brain tissue and induces high intracranial pressure, leading to primary brain injury (PBI) [10]. A previous report has shown that craniotomy and minimally invasive endoscopic procedures for hematoma evacuation effectively limit PBI following ICH [11]. However, early surgery shows no amelioration for the long-term outcomes compared with initial conservative treatment [12]. Besides, it has been shown that debris and degradation products of red blood cells and hemoglobin mediate a string of injured events after ICH, such as brain edema, blood–brain barrier (BBB) damage, inflammatory response, demyelination, axonal damage, and neuronal death, which is defined as secondary brain injury (SBI) [3, 13–17]. In clinical research, more than 70% of patients with ICH live with cognitive impairment, and over 30% suffer severe movement dysfunction [18, 19]. Therefore, more studies on ICH have recently paid more attention to ICH-induced SBI to explore promising therapeutic strategies.

Mesenchymal stem cells (MSCs) are regarded as hopeful seed cells and utilized widely in preclinical research, especially in kinds of stroke diseases, because of their favorable properties, including safety, ease of cultivation, and weak immunogenicity [20–22]. Li et al. found that the administration of MSCs can alleviate neurological deficits after ICH [8]. The implanted MSCs have a limited effect in repairing the injured tissue because of their poor survival rate in brain disease [23]. Numerous studies have shown that traditional Chinese medicine (TCM), such as single herbs and their extracts, possesses positive functions on the differentiation and proliferation of MSCs. Quercetin, as a flavonoid, extensively exists in varieties of herbs and can facilitate neuronal function in repairing brain damage by inhibiting the inflammatory response and apoptosis [24]. Quercetin can regulate cell proliferation, migration, autophagy, and other biological functions, thus exerting anti-inflammatory effects [23, 24]. However, quercetin’s poor water solubility remarkably restricts its biomedical applications. Nathupakorn et al. have synthesized the iron-quercetin complex (IronQ), which not only can enhance peripheral blood mononuclear cells (PBMCs) growth but also be used as a tracking probe in MRI [25, 26]. If IronQ could be used to improve MSCs growth to enhance their therapeutic effects after ICH, it would be a promising therapy for relieving brain injury.

Compared with the adaptive immune system, various pathogens can be recognized by innate immune receptors, playing an essential role in regulating neuroinflammation [27–29]. As an essential innate immune cell of the central nervous system (CNS), microglia are usually regarded as the macrophages of brain tissue. Many studies suggest that ICH-induced inflammation exacerbates damage to brain tissue. For instance, inflammatory factors released by activated microglia can further damage the brain tissue, while chemoattractant peripheral inflammatory cells infiltrate the CNS, aggravating local inflammatory response [7]. Microglia is the primary source of inflammatory factors such as IL-6 and TNF-α after ICH. Macrophage-inducible c-type lectin (Mincle) is a newly defined non-typical c-type lectin receptor (innate immune receptor), which is mainly expressed in microglia/macrophages [30]. Stimulated by certain fungi, mycobacterium tuberculosis, and necrotic cells, it binds to the associated ligand (SAP130), phosphorylating downstream effector spleen tyrosine kinase (syk) and activating card 9-dependent cascade signals [30, 31]. The content of card 9 is directly related to the effect of the immune response, and the card9-bcl10-malt1 complex can activate its downstream target, nuclear factor-kappa B (NFκB) [32, 33]. Activation of the NFκB signal increases the expression levels of inflammatory factors, suggesting that Mincle is a crucial target for regulating microglia/macrophage cell polarization in traumatic brain injury (TBI) [30], subarachnoid hemorrhage (SH), and IS [31–33]. Nevertheless, the underlying mechanism for the Mincle signal axis attending ICH-induced pathological process needs further exploration.

In this study, we investigated the effects of MSCs with IronQ on the neurological deficits score, brain water content (BWC), protein expression of neuron-specific nuclear (NeuN, a marker of neurons), glial fibrillary acidic protein (GFAP, a marker of astrocytes), myelin basic protein (MBP, a marker of myelin), as well as the inflammatory factors in mice with ICH and Mincle, and its downstream was also investigated to explore the neuroprotective effect, anti-inflammatory function, and the potential mechanisms of combined treatment.

## Materials and methods

### Chemicals

The IronQ complex was synthesized according to our previous research work [25]. Shortly, 0.0050 mol of quercetin hydrate (Sigma, USA) was supplemented to 500 mL HPLC-methanol (Sigma, USA) with continued stir until complete dissolution of quercetin hydrate, showing the yellow solution. The pH of the solution was then slowly adjusted to 12 based on the addition of NaOH solution to obtain a deprotonated form of quercetin. 0.0025 mol Iron (III) chloride (Sigma, USA) in 500 mL ultrapure water (up water) was then blended with the above solution until the solution color turned to dark yellow, which was followed by incubation with a continuous stirring for 2 h at 60 °C. And then the final solution was purified and evaporated to dryness. The dark powder product was collected and stored it away from light at room temperature (RT).

### Animals

**Fig. 1:**
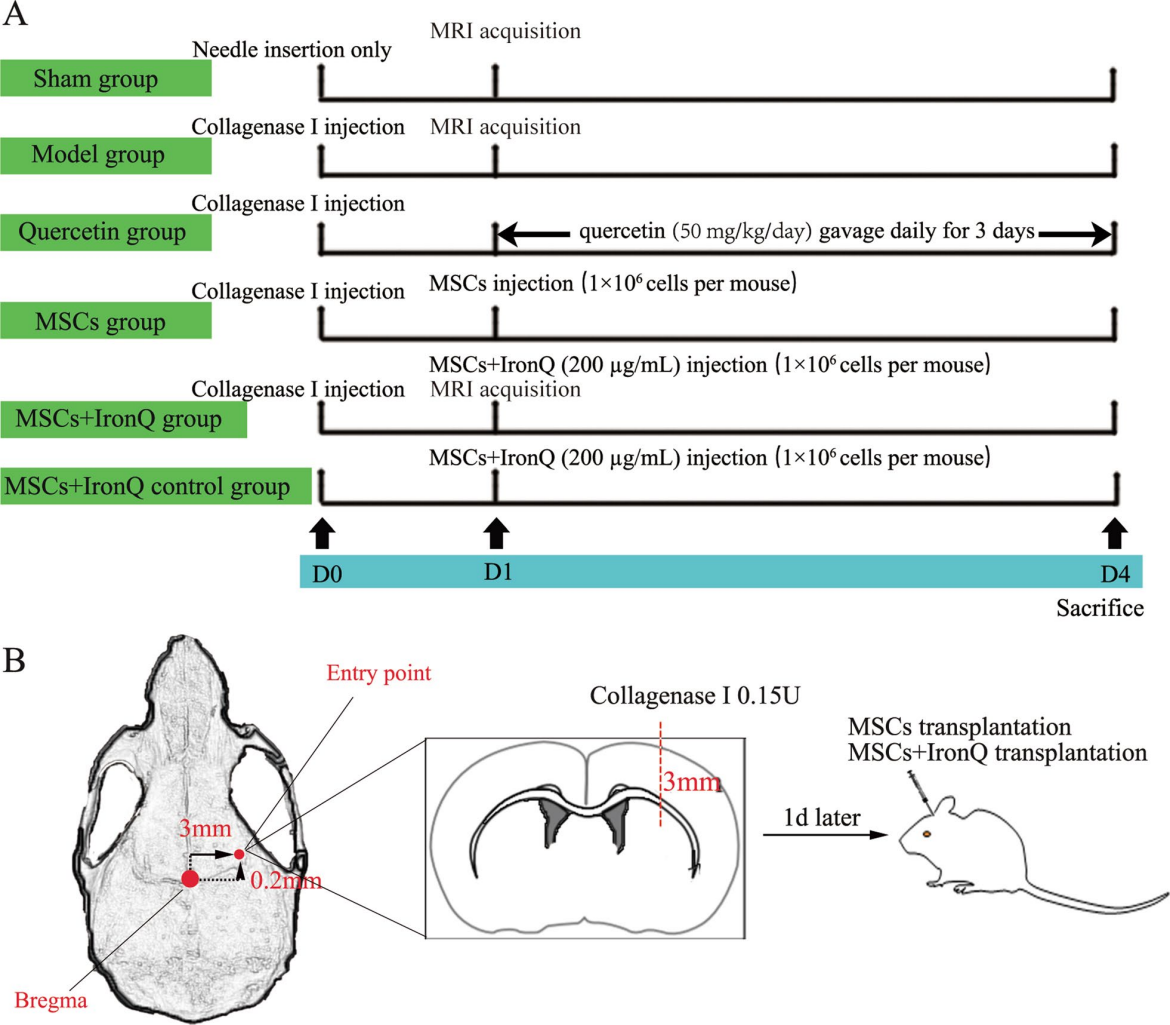
Conceptual diagram of the experimental protocols. **A** Brief schedule of experimental mouse grouping and treatment strategies and experimental procedures. **B** Schematic diagram of ICH mice model transplanted with MSCs and MSCs + IronQ

One hundred wild-type C57BL/6 male mice, 8–9 weeks old, weighed between 22 and 25 g, were purchased from Chongqing Tengxin Biotechnology Co., Ltd (Chongqing, China). They were housed according to 5 mice per cage for acclimatization under the same animal care facility for three days. The animals were monitored by the research unit once daily. If the mice got the following symptoms, including lethargy, shortness of breath, skin discoloration or irregularity, enlargement of lymph nodes, and solid visible tumors under the subcutis, mice were euthanized directly. These morbid mice were documented as “adverse events”. The procedure for using the animals followed the Guidance Suggestions for the Care and Use of Laboratory Animals formulated by the Ministry of Science and Technology of China. Besides, it was approved (Approval No. 20211122–040) by the Animal Ethical Committee of the Animal Center of Southwest Medical University (Luzhou, Sichuan). The optimized experimental procedures minimized the number of experimental animals and alleviated their suffering. The mice (90 mice) were blindly and randomly divided into six groups (*n* = 15 mice per group): the sham operation group (Sham), the model group (Model), the quercetin gavage group (Quercetin), the MSCs transplantation group (MSCs), MSCs transplantation combined with IronQ group (MSCs + IronQ), and MSCs + IronQ control group, and then assigned to the corresponding experimental procedures (Fig. 1A).

### Intracerebral hemorrhage mice model

Mice were anesthetized intraperitoneally with 40 mg/kg of 1% pentobarbital sodium and fixed in a stereotaxic apparatus (supine position) with the anterior and posterior bregma kept at the same level. Incise the scalp sagittally about 1 cm and expose the anterior fontanelle with 30% H_2_O_2_. The mixture containing 1μL 0.15 U/μL collagenase I (C8140, Solarbio, China) and supplementary 0.9% normal saline was extracted with a 1μL microsyringe. Made an opening (1 mm) in the right skull, 2.5 mm lateral and 0.2 mm anterior. The needle was attached to the stereotaxic device, inserted it into the caudate nucleus (3 mm deep from the hole), and injected the mixture slowly (Fig. 1B). After injection, the drilled hole was closed with bone wax, and the scalp was sutured. The ICH mice were randomly and blindly separated into four ICH experimental groups. Among them, the mice in the quercetin group for intragastric administration with quercetin (50 mg/kg/day) after 24 h when successfully modeled were performed, referring to the previous study [23, 24]. The specific conceptual illustrations of the experimental protocols for the MSCs and MSCs + IronQ are given in Fig. 1. The mice in the sham operation group performed the same operation without the injection of the mixture, and the mice in the MSCs + IronQ control group were given the injection of the complex of MSCs with IronQ. After successfully modeled and treated with MSCs transplantation combined with IronQ, the sham operation, model, and MSCs + IronQ groups were visualized using 3.0 T MRI. Mice in all groups were euthanized by an overdose of intraperitoneal injection of 40 mg/kg of 1% pentobarbital sodium for harvesting the brain samples in the following experiments.

### Neurological Function Assessment by modified Neurologic Severity Score (mNSS) Test

The mNSS was operated as previously reported [34]. Neurologic deficits were performed by evaluating abnormal movements, including motor, sensory, and reflex deficits, at 24 h after ICH induction using the mNSS system (18-point neurological deficit scale, normal score, 0; maximal score, 18), which is extensively administrated to evaluate the degree of ICH-induced nerve damage. The higher score represents the more severe neurological damage. After 24 h of the ICH model constructed by the right caudate nucleus injection of collagenase, mice were evaluated and scored blindly. The criteria are 13–18 points, representing severe damage; 7–12 points, representing moderate damage; 1–6 points, representing slight damage. When assessing neurological function, one point is awarded for the inability to complete the test or lack of relevant reflexes. That is to say, the more elevated the score, the more severe the injury. This study used ICH mice with moderate damage for the subsequent experiments.

### Extraction, Passage, and Identification of MSCs

Obtaining the MSCs is followed by the methodology as previously described [35]. Briefly, the femurs and tibias were isolated from the male healthy C57BL/6 mice (total = 10 mice) on the asepsis condition after intraperitoneal injection anesthesia. Then, the muscles were carefully removed on the bone with a scissor. Their medullary cavities were several washed by Dulbecco’s modified eagle medium (DMEM, Gibco, USA) to acquire a uniform suspension with mixed cells. Centrifuged the cells and removed the red blood cells with red blood cell lysate (R1010, Solarbio, China). The cells were collected and cultured in DMEM medium supplemented with 10% fetal bovine serum (FBS, Gibco, USA) and 1% penicillin/streptomycin (C0222, Beyotime Biotechnology, China) at 37 °C and 5% CO_2_ in a humidified environment. Cells from the third passage were harvested, and 1 × 10^6^ single cells were incubated with the primary antibodies against CD90, CD29, and CD45 (Bioscience, USA) for 30 min at 37 °C. Flow cytometry accomplished cell identification (BD FACSCanto II, BD Biosciences, USA).

### MTT assay

The effect of IronQ on cell proliferation was demonstrated by utilizing the MTT assay. Shortly, MSCs were seeded at a density of 2 × 10^3^ cells/well in a 96-well plate and cultivated in DMEM (Gibco, USA) with 10% FBS (Gibco, USA) and 1% penicillin/streptomycin (C0222, Beyotime Biotechnology, China). After 24 h of incubation, MSCs were treated with IronQ at different concentrations (0, 200, 600, 1000, and 2000 μg/mL) and incubated for 24 h. The cells were washed twice with PBS and added 200 μL DMEM + 20 μL 5 mg/mL MTT (Amresco, USA) solution to continue incubation for 4 h at 37 °C. Then, the medium was discarded and replaced with 200 μL DMSO to dissolve the MTT formazan crystals. The absorbance at 490 nm of the solution was measured using the SYNERGY2 microplate reader (BioTek, USA).

### IronQ labeling of MSCs and determination of labeling efficiency by Prussian blue

MSCs (1 × 10^6^ cells, 5 mL) were seeded in a 6-well plate with the DMEM (Gibco, USA) accompanied by 10% FBS (Gibco, USA) and 1% penicillin/streptomycin (C0222, Beyotime Biotechnology, China). After MSCs adherence, IronQ solution (1000 μg/mL, IronQ dissolved with sterile up water) was added to the six-well plate at diverse concentrations (0, 50, 100, 200, and 400 μg/mL). Cells were then continued to be cultivated for 2 days at 37 °C with 5% CO_2_ in a humidified incubator. After the indicated time, the cells were washed 3 times with PBS to remove the unbound IronQ. Prussian blue stain confirmed the intracellular uptake of IronQ by MSCs. In brief, the cells were fixed with 4% paraformaldehyde for 20 min in a humidified incubator at 37 °C. Then, the fixation reagent was replaced with 1 mL of pearl blue reaction solution (G1422, Solarbio, China) in each well and re-incubated for 30 min. After that, cell morphology and the blue-stained positive cells were visualized and photographed using a Nikon-inverted fluorescence microscope (ECLIPSE Ti, Nikon, Japan) with a Nikon DS-Ri2 camera, and images were captured using Nikon software NIS-Elements F.4.30.01, at magnification 100× based on an objective lens of 10× and eyepiece of 10×. The images were processed using image processing software such as Adobe illustrator 2021.

### Transplantation of MSCs and MSCs combined with IronQ

Relative experiments were performed by MSCs and the combination of MSCs with IronQ at passages 3–6. The cells were harvested and adjusted the cell concentration to 5 × 10^7^ cells/mL. Extracted cell suspension (20 μL) by a microsyringe was injected into the point (position: the right of bregma: 3 mm; front of bregma: 0.2 mm; depth: 3 mm) of mouse brains for MSCs, MSCs + IronQ, and MSCs + IronQ control groups, respectively, at 2 μL/minute 24 h after successfully modeling. Then, the pinhole was sealed with bone wax and sutured and sterilized the skin.

### MRI of MSC^IronQ^ transplant in ICH Mice

Mice were anesthetized as the above modeling method throughout the MRI examination. MRI was performed using a 3-Tesla MRI scanner (Siemens, Germany). T1 fast spin-echo (repetition time/echo time = 500/11 ms) was used to acquire a field of view of 16 × 16 mm^2^, matrix of 256 × 256, and 0.75-mm thick for coronal, axial, and sagittal plane. The single image was preserved as 1019 × 602-pixel picture for T1 lesion evaluation.

### Hematoxylin–eosin Staining and Nissl staining

Perfusion-fixed brain tissue was further fixed by soaking overnight in 4% formaldehyde, as previously described [36]. The tissue was then dehydrated through a series of graded alcohols followed by xylene for 30 min each. The brain tissue was then embedded in paraffin and cut into 4-µm-thick sections frontally using a microtome (RM 2245, Leica, Germany). Tissue sections were dewaxed and rehydrated for staining. Hematoxylin–eosin (HE) staining and Nissl staining were performed, referring to standard procedures and the manufacturer’s instructions. Brain tissue slides were observed under a Leica optical microscope (DM500, Leica, Japan) equipped with a Leica ICC50W camera, and images were magnified 200 × based on an objective lens of 20 × and eyepiece of 10 × using Leica Software LAS X. The images were processed using image processing software such as Adobe illustrator 2021.

### BWC Examination

BWC was measured as previously reported [37]. In short, brain samples were harvested from anesthetized animals and immediately weighed to determine the wet weight via a precise electronic scale. After drying at 100 ℃ in the thermostat for 24 h, brain samples were reweighed to get their dry weight. The formula of BWC is calculated as [(wet weight – dry weight)/wet weight] × 100%.

### Immunofluorescence

Immunofluorescence staining was carried out as previously reported [37]. Under deep anesthesia of the mice, the heart was perfused with 0.9% normal saline and 4% paraformaldehyde with 0.01 M phosphate-buffered saline (PBS, pH7.4) successively. Brain samples were collected, fixed in 4% paraformaldehyde for 24 h at 4 °C, and dehydrated in the 30% sucrose solution (prepared in PBS) for an additional 24 h until the samples sank to the bottom of the sucrose solution. The samples in optimal cutting temperature compound (OCT) were sectioned coronally for 4 μm at the level of basal ganglion by using the freezing microtome (CM1950, Leica, Germany). After washing three times with PBS, tissue sections were blocked for 1 h at RT with 5% BSA and incubated with primary antibodies, including mouse anti-IL-6 (sc32296, Santa Cruz, USA, dilution 1:100), mouse anti-TNF-α (sc52746, Santa Cruz, USA, dilution 1:100), mouse anti‐Mincle (sc390806, Santa Cruz, USA, dilution 1:100), rat anti‐F4/80 (sc52664, Santa Cruz, USA, dilution 1:100), rabbit anti-GFAP (16825-1-AP, Proteintech, USA, dilution 1:100), rabbit anti-MBP (10458-1-AP, Proteintech, USA, dilution 1:100), and rabbit anti-NeuN (12943, CST, USA, dilution 1:100) overnight at 4 °C. The following day, tissue sections were washed three times with PBS for 5 min each and then treated with Alexa Fluor® 555 conjugated anti‐mouse secondary antibody (A21424, Life Technologies, USA, dilution 1:500), Alexa Fluor™ 555 conjugated anti‐rabbit second antibody (A21428, Invitrogen, USA, dilution 1:1000), Alexa Fluor™ 488 conjugated anti‐mouse secondary antibody (A11001, Invitrogen, USA, dilution 1:500), and Alexa Fluor™ 488 conjugated anti‐rabbit secondary antibody (A11034, Invitrogen, USA, dilution 1:500) incubation for 1 h at RT. Images were recorded by a Leica Fluorescence orthotopic microscope (DM4B, Leica, Germany) equipped with a Leica DMC6200 camera using the software LAS X, at a magnification of 200 × based on an objective lens of 20 × and an eyepiece of 10 × . The images were processed using image processing software such as Adobe illustrator 2021.

### Western Blot

Mice were decapitated under deep anesthesia, and brain samples were quickly collected and separated into ipsilateral brain hemispheres. Proteins were uniformly extracted with RIPA lysis buffer (Beyotime, China) and incubated at 13,000 rpm at 4 °C, and the supernatant was collected. Then, the total protein concentration in each sample group was determined by bicinchoninic acid (BCA) protein assay (Beyotime, China). Equal amounts of protein samples (50 μg) were blotted to nitrocellulose (NC) membranes by sodium dodecyl sulfate–polyacrylamide gel electrophoresis (SDS-PAGE). Membranes were then incubated with the following primary antibodies against IL-6 (sc32296, Santa Cruz, USA, dilution 1:1000), TNF-α (sc52746, Santa Cruz, USA, dilution 1:1000), NeuN (12943, CST, USA, dilution 1:1000), MBP (10458-1-AP, Proteintech, USA, dilution 1:1000), Mincle (sc390806, Santa Cruz, USA, dilution 1:1000), syk (13198, CST, USA, dilution 1:1000) and p-syk (2710, CST, USA, dilution 1:1000), NFκB-p65 (sc8008, Santa Cruz, USA, dilution 1:1000), p-NFκB-p65 (3033, CST, USA, dilution 1:1000), and GAPDH (Abcam, dilution 1:10,000) overnight at 4 °C. After incubated with Alexa Fluor™ 790 conjugated anti‐mouse secondary antibody (A11359, Invitrogen, USA, dilution 1:3000), Alexa Fluor™ 680 conjugated anti‐mouse secondary antibody (A21109, Invitrogen, USA, dilution 1:3000), and Alexa Fluor™ 680 conjugated anti-rat secondary antibody (A21096, Invitrogen, USA, dilution 1:3000) for 2 h at RT, the blots were exposed under a Far Infrared Laser Imaging System (Amersham Typhoon, USA). Protein content was analyzed by the corresponding amount of GAPDH using Image J software.

### Real‐Time Polymerase Chain Reaction (qPCR) Analyses

Total RNA was extracted from mouse brain tissue using TRIzol reagent (11596026, Invitrogen, USA) according to the manufacturer’s protocol. One μg of RNA was removed from each sample and reverse-transcribed into complementary DNA for qPCR using HiScript III RT SuperMix (+ gDNA wiper) (No. R323-01, Vazyme, China). A fluorescent dye ChamQ Universal SYBR qPCR Master Mix (No. Q711-02/03, Vazyme, China) was used for the reaction in Mastercycler ep Realplex2 real‐time PCR system (Eppendorf, Germany). Specific primers for qPCR were purchased from Sangon Biotech (Shanghai) Co., Ltd, as shown in Table 1.

**Table 1:** List of primers sequence

Gene Name	Primer sequence (5′–3′)	Product Length
IL-6	F: AAAGAGTTGTGCAATGGCAATTCT	24
	R: AAGTGCATCATCGTTGTTCATACA	24
TNF-α	F: CATCTTCTCAAAATTCGAGTGACAA	25
	R: TGGGAGTAGACAAGGTACAACCC	23
Mincle	F: ACCAAATCGCCTGCATCC	18
	R: CACTTGGGAGTTTTTGAAGCATC	23
IL-1β	F: TGCCACCTTTTGACAGTGATG	21
	R: AAGGTCCACGGGAAAGACAC	20
GAPDH	F: CGGAGTCAACGGATTTGGTCGTAT	24
	R: AGCCTTCTCCATGGTGGTGAAGAC	24

### BV2 cell culture and experiments

Murine BV2 microglial cells were purchased from China Infrastructure of Cell Line Resource (Beijing, China) and cultured in DMEM (glucose containing 4.5 g, Gibco, Invitrogen) spiked with 10% FBS (Gibco, Invitrogen) and 1% penicillin/streptomycin (C0222, Beyotime Biotechnology, China) in a 5% CO_2_ humidified incubator at 37 °C. The cells were incubated with LPS (200 ng/mL) and treated with quercetin (Q), the conditioned medium of MSCs (M), and the conditioned medium of MSCs + IronQ (M + Q) for 6 and 24 h to check mRNA and protein expression levels, respectively. Then, the trehalose-6,6-dibehenate (TDB) (66758-35-8, Invivogen, USA), a Mincle agonist, was utilized to activate Mincle including its mRNA and protein expression. According to the manufacturer’s illustration, TDB was dissolved and made the concentration at 1 mg/mL (DMSO/PBS:1/9) for the store. BV2 cells were incubated with LPS (200 ng/mL) and TDB (70 μg/mL) in the absence of M + Q for 6 and 24 h. Then, BV2 cells were collected for the following qPCR and western blot experiments.

### Statistical analysis

Parametric data were analyzed by GraphPad Prism 8 software and presented as the mean ± standard error of the mean (SEM). Statistical differences among multiple groups were analyzed by one-way analysis of variance (ANOVA), and *P* < 0.05 was considered statistically significant.

## Results

### The Efficient MSCs labeling and IronQ-labeled MSCs tracking in the ICH model using T1-weighted MRI

**Fig. 2:**
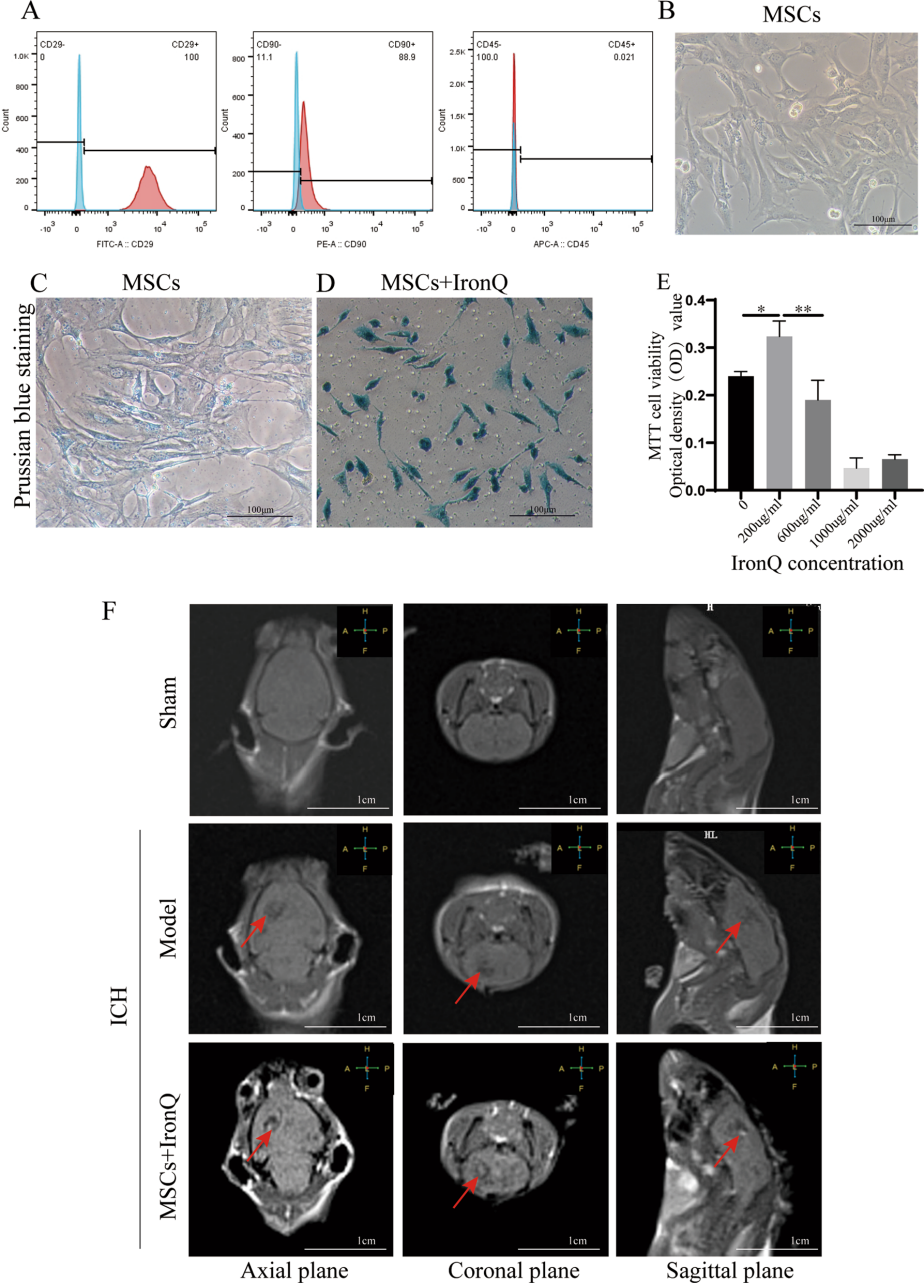
The images of MSCs and the IronQ-labeled MSCs from an inverted phase-contrast microscope and 3.0 T MRI. **A** Identification results of MSCs by flow cytometry. **B** MSCs at passages 3–6 grew in a cluster shape. Scale bar = 100 μm. **C, D** The images of MSCs incubated without (**B**) and with (**C**) IronQ for 24 h were checked by Prussian blue staining. Scale bar = 100 μm. **E** MTT assay of IronQ for MSCs. **F** T1W images of mice brains in the coronal plane, axial plane, and sagittal plane of the sham operation, model, and MSCs + IronQ groups. Arrow indicated a hemorrhage (dark) area and IronQ-labeled MSCs (a white spot) in the hemorrhage (dark) area of the ipsilateral striatum in the brain tissue of ICH mice. Scale bar = 1 cm

Mice tibia and femur bone marrow were extracted and cultured to the third passage and identified by their positive markers CD29 and CD90 and negative marker CD45 (Fig. 2A). MSCs in passages 3–6 showed cluster growth (Fig. 2B and Additional file 1). After incubating MSCs with IronQ (200 μg/mL) at 37 °C for 24 h in a humified CO_2_-incubator, the labeling efficiency of IronQ for MSCs was identified by Prussian blue staining, and the labeled cells displayed blue color (Fig. 2C-D and Additional file 1). MTT assay indicated that the concentration of 200 μL/mL IronQ improved the MSCs proliferation significantly (Fig. 2E). Figure 2F shows the T1-weighted MRI images of mice brains in sham operation, model, and MSCs + IronQ groups. The results displayed that the apparent hemorrhage (dark area) in the right caudate nucleus of mice in the model group can be observed in three MRI planes, including the coronal, axial, and sagittal planes, compared with the mice in the sham operation group. After transplantation of MSCs + IronQ, MSCs labeled with IronQ were observed as a white spot in the hemorrhage region of the ipsilateral striatum of mice brain under MRI.

### MSCs and IronQ combined treatment attenuated the neurological deficits and protected the brain parenchyma and neurons survival after ICH

**Fig. 3:**
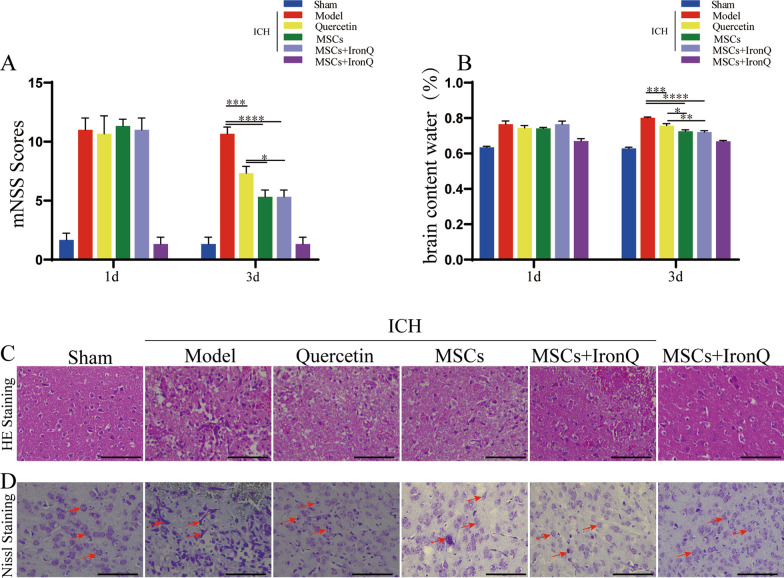
MSCs + IronQ transplantation attenuated the neurological deficits and protected the brain parenchyma and neurons in mice ICH. **A** The results of mNSS displayed the points in each group of mice on day 1 and day 3 (*n* = 5 for each group; * P < 0.05, ***P < 0.001, and ****P < 0.0001). **B** BWC of brain samples in each group (*n* = 5 for each group; *P < 0.05, **P < 0.01, ***P < 0.001, and ****P < 0.0001). **C** HE staining of brain samples was performed in the ipsilateral striatum of mice brain tissue in each group. Scale bar = 100 μm. **D** Nissl staining of brain samples was performed in the ipsilateral striatum of mice brain tissue in each group. Scale bar = 100 μm

After 24 h of the collagenase-induced ICH model, the mNSS system (18 points) was used to assess neurological deficits in mice with ICH. Figure 3A displays that the collagenase-induced ICH mice model has a similar neurological deficits level among the experimental groups (*P* > 0.05). After giving corresponding treatment to the ICH mice in each group, the neurological deficits score in the three treatments (quercetin, MSCs, and MSCs + IronQ) groups has remarkable improvement. The combined treatment significantly improved the neurological deficits more than the mice in the quercetin and MSCs groups (*P* < 0.05). Meanwhile, consistent results were also confirmed in measuring the BWC (Fig. 3B). Specifically, 24 h after ICH, BWC was enhanced significantly compared with the mice in the sham operation group and MSCs + IronQ control groups (*P* < 0.05). Three treatments can decrease the BWC compared with the model group. However, MSCs and the IronQ-labeled MSCs transplantation can attenuate the BWC more significantly compared with the quercetin gavage, and there is no significance between MSCs and MSCs + IronQ groups. Simultaneously, Fig. 3C displays HE staining in the ipsilateral striatum of mice brain tissue in each group. There were no pathological changes, the neuropil was intact, and the pyramidal neurons had healthy nuclei in the mice brain tissue sections in the sham operation group. However, obvious pathological changes in the perihematomal region of the brain were observed in the model group. Defective neuritis with vacuolation, parenchymal loss, granulovacuolar neurodegeneration, neuronal atrophy, and reactive gliosis existed. Surprisingly, compared with quercetin and MSCs therapy, MSCs + IronQ treatment preserved the normal neuropil architecture and reduced neurodegeneration and reactive gliosis.

Nissl staining revealed ICH-mediated brain damage in the ipsilateral striatum of mice brain tissue, as loss of Nissl substance indicated neuronal injury (Fig. 3D). The results showed multiple blue-colored Nissl bodies were present in the sham operation group pyramidal neurons of brain tissue sections. The Nissl bodies displayed obvious pyknosis and swallow section phenomenon, which was further reduced in three treatment groups. MSCs + IronQ treatment group revealed an increase in the number of neurons stained with Nissl staining compared with quercetin and MSCs treatment groups, indicating its neuroprotective effect.

### MSCs and IronQ combined treatment increased the protein expression of NeuN, MBP, and GFAP in ICH Mice

**Fig. 4:**
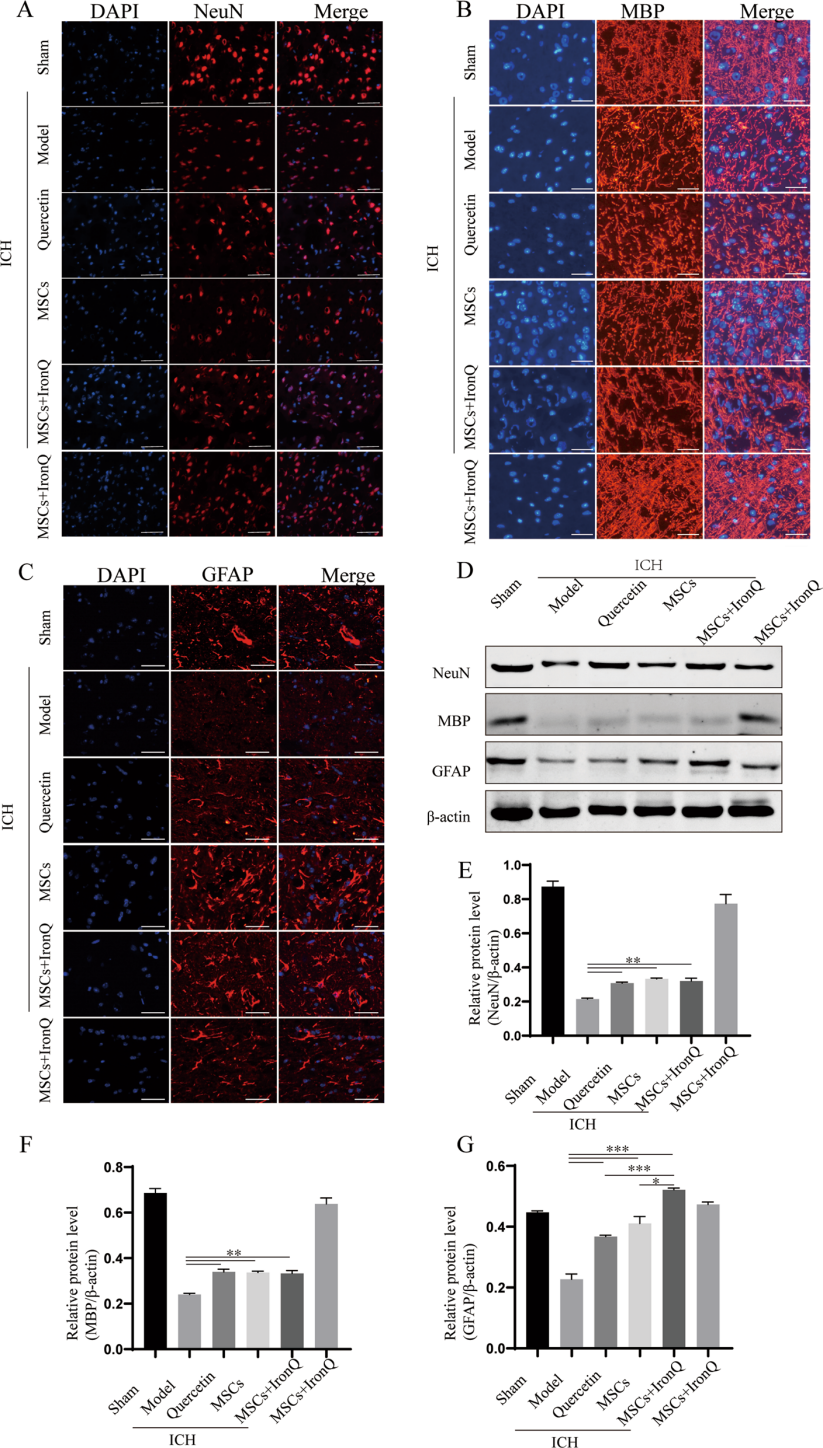
MSCs and IronQ combined treatment increased NeuN, MBP, and GFAP protein expression in mice with ICH (**A-C**) Immunofluorescent results displayed the protein expression levels of NeuN, MBP, and GFAP in the ipsilateral striatum of mice brain tissue after ICH (*n* = 5 for each group). Scale bar = 100 μm. **D-G** The results and analyses of western blot for NeuN, MBP, and GFAP protein expression (*n* = 5 for each group; **P < 0.05, **P < 0.01, ***P < 0.001)

Figure 4 displays the results and analyses of immunofluorescence and western blot of NeuN, MBP, and GFAP protein expression in the ipsilateral striatum of mice brain tissue in different groups. Specifically, Fig. 4A-C shows that the NeuN, MBP, and GFAP staining in the brain tissue of the sham operation group were evenly distributed and the expression levels of NeuN, MBP, and GFAP decreased after ICH. In contrast, the positive NeuN and MBP staining increased in the treatment groups (Fig. 4A-C). The western blot results verified consistent results with the immunofluorescence (Fig. 4D-G and Additional file 2), displaying that NeuN, MBP, and GFAP expression in the sham operation group was higher than in the model group. However, after exerting the corresponding treatment in mice with ICH of each group, the protein expression levels of NeuN, MBP, and GFAP increased significantly compared with the mice in the model group. Moreover, there was no significance in the NeuN and MBP protein expression among the three treatment groups, and only the GFAP protein expression in the MSCs + IronQ group was higher than that in the quercetin (*P* < 0.001) and MSCs (*P* < 0.05) groups.

### MSCs and IronQ combined treatment decreased the inflammatory response in mice with ICH

**Fig. 5:**
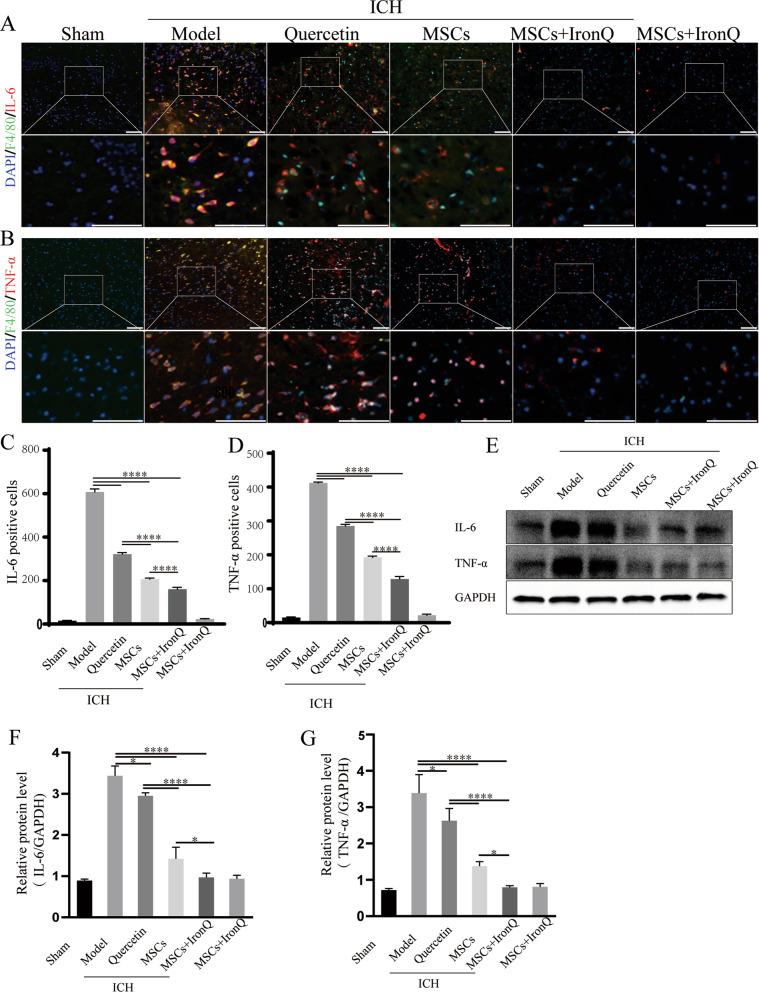
MSCs and IronQ combined treatment decreased the inflammatory response in mice with ICH. **A, B** The results of double immunofluorescence displayed co-localization for F4/80 co-expressing with IL-6 and TNF-α in the ipsilateral striatum of the mice brain tissue section of each group. Respective change in color showing co-localized signals in merged figures corresponds to Red + Green = Yellow; Red + Blue = Magenta. Scale bar = 100 μm (top panel). The white box areas are shown at higher magnification in the bottom panel. **C-D** showed the statistical graph of positive IL-6 and TNF-α staining cells (*n* = 5 for each group; ***P < 0.001, and ****P < 0.0001). **E–G** The results and analyses of western blot of IL-6 and TNF-α protein expression in each group (*n* = 5 for each group; *P < 0.05, and ****P < 0.0001)

Figure 5A-D shows the double immunofluorescence of F4/80 (the microglia marker) with IL-6 and TNF-α in the ipsilateral striatum of mice brain tissue in each group. The sham operation group did not detect the co-expression staining of F4/80 with IL-6 and TNF-α. However, co-expression of F4/80 with uniformly distributed IL-6 and TNF-α staining was observed throughout the mice brain tissue of the model group. Interestingly, we found that microglial IL-6 and TNF-α staining decreased in three treatment (quercetin, MSCs, and MSCs + IronQ) groups. In addition, the number of co-expression of F4/80 with IL-6 and TNF-α reduced in MSCs + IronQ compared with the other two treatment groups. Figure 5E-G and Additional file 3 display the western blot results of the inflammatory factors IL-6 and TNF-α. The results showed that compared with the sham operation group, the protein expression of IL-6 and TNF-α increased more significantly after ICH (*P* < 0.0001). MSCs transplantation downregulated their expression levels more significantly than quercetin gavage (*P* < 0.0001), but the combined treatment decreased their expression levels remarkably compared with MSCs treatment (*P* < 0.05).

### MSCs and IronQ combined treatment regulated the Mincle/syk signaling pathway for the improvement of ICH outcomes

C-type lectin-like receptors (CLRs) represent a family of transmembrane pattern recognition receptors, showing a high expression on myeloid cells. Dysfunction of these receptors may induce the degradation of inflammatory illnesses, such as IS and multiple sclerosis. In particular, Mincle is a key member of CLRs and is fully expressed in macrophages, where it moderates damaged cell-mediated innate immune responses in various pathological changes [38]. Following activation, Mincle and its downstream syk increase inflammatory cytokine production, such as TNF-α and IL-6, by regulating the NFκB signal in brain diseases [32, 38–40].

**Fig. 6:**
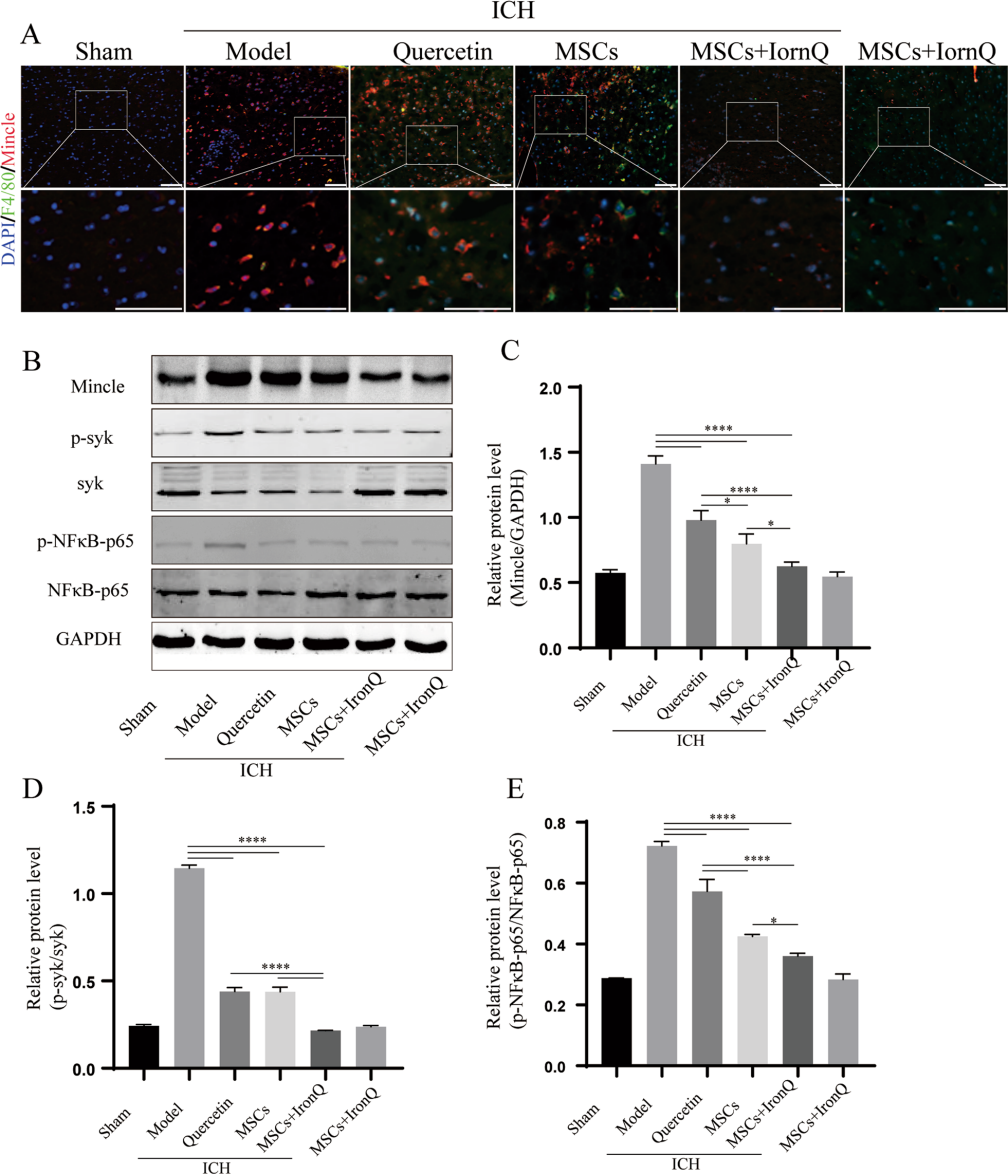
MSCs and IronQ combined treatment regulated the Mincle/syk signaling pathway to improve ICH outcomes. **A** The results of double immunofluorescence displayed co-expression of F4/80 with Mincle in the ipsilateral striatum of mice brain tissue in each group (*n* = 5 for each group). Respective change in color showing co-localized signals in merged figures corresponds to Red + Green = Yellow; Red + Blue = Magenta. Scare bar = 100 μm (top panel). The white box areas are shown at higher magnification in the bottom panel. **B-E** The results and analyses of western blot of Mincle, p-syk, and p-NFκB-p65 protein expression in mice with ICH in each group (*n* = 5 for each group; *P < 0.05, and ****P < 0.0001)

In this study, we utilized immunofluorescence and western blot to explore the protein expression levels mentioned above. Specifically, Fig. 6 shows the double immunofluorescence of F4/80 with Mincle and the western blot of Mincle and its downstream in mice with ICH in each group. The co-expression of Mincle and F4/80 was detected via double immunofluorescence in the ipsilateral striatum of mice brain tissue (Fig. 6A). In mice brain sections of the sham operation group, fewer positive co-expression cells of F4/80 with Mincle were detected, and their co-expression cells increased in mice brain sections of the model group. There was no significance between the MSCs and quercetin groups in the co-expression of F4/80 with Mincle-positive cell levels. Conversely, compared with the other two treatment groups, the co-expression of F4/80 with Mincle-positive cells was downregulated in mice brain sections of the MSCs + IronQ group (Fig. 6A).

Moreover, Mincle and its downstream (p-syk and p-NFκB-p65) protein expression were detected by western blot. The immunoblotting results displayed that Mincle, p-syk, and p-NFκB-p65 protein expression increased obviously in mice brain tissue of the model group, and three different treatments (quercetin, MSCs, and MSCs + IronQ) evenly downregulated their expression levels (*P* < 0.0001). MSCs transplantation decreased Mincle (*P* < 0.05) and p-NFκB-p65 (*P* < 0.0001) remarkably compared with quercetin gavage treatment, and no significance for p-syk can be seen between the MSCs and quercetin groups. Positively, the combined treatment exerted the downregulation effects of Mincle and its downstream protein expression significantly compared with the quercetin and MSCs groups (*P* < 0.05) (Fig. 6B-E and Additional file 4).

### Conditioned medium of MSCs with IronQ reduced the mRNA and protein expression levels of mincle and inflammatory factors in LPS-induced BV2 cells

**Fig. 7:**
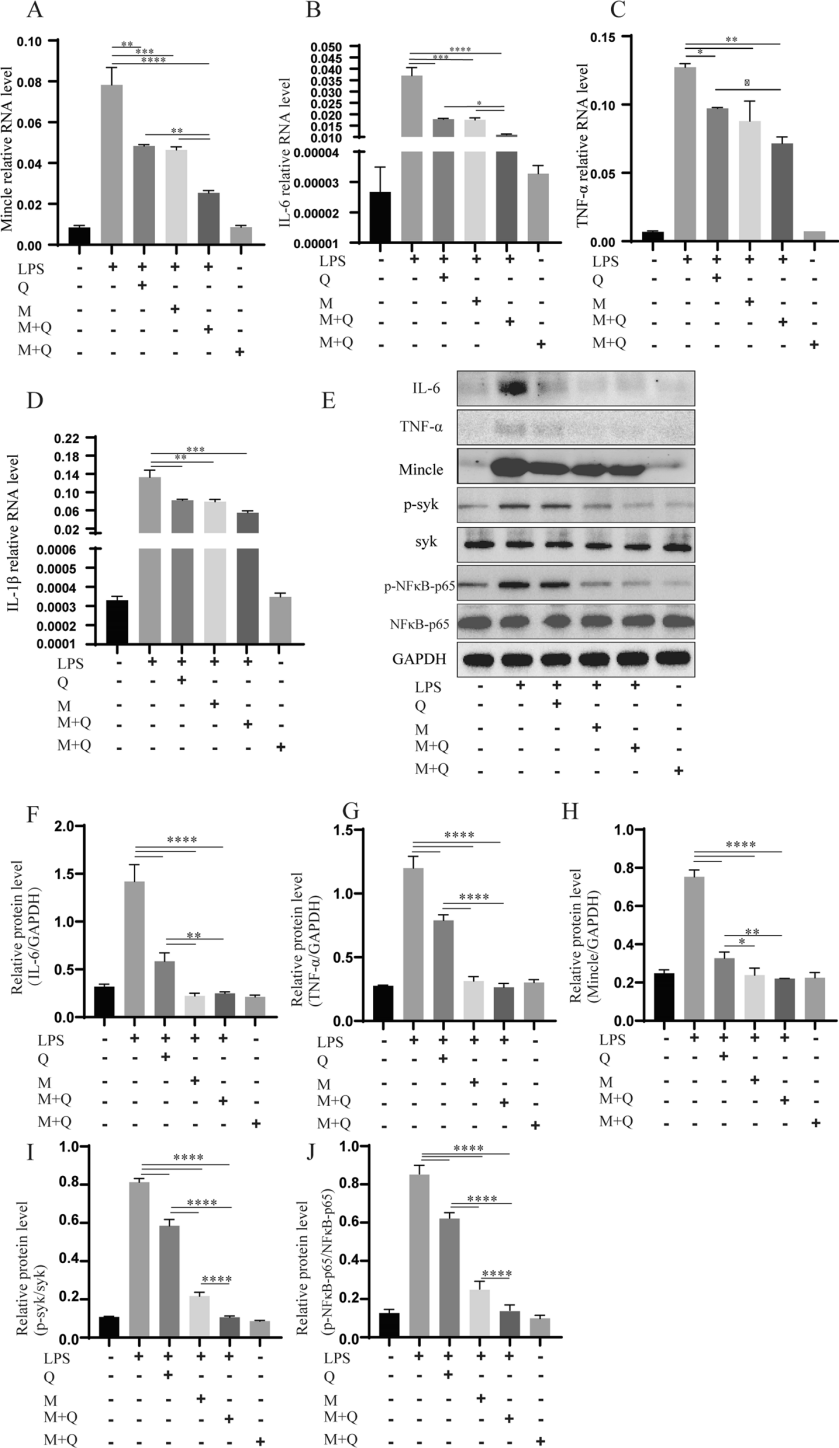
The results and analyses of qPCR and western blot revealed that the conditional medium of MSCs with IronQ reduced mRNA and protein expression of Mincle and inflammatory cytokines. **A–D** The presentation of graphs for **A** Mincle, **B** IL-6, **C** TNF-α, and **D** IL-1β mRNA relative expression in different cells groups after 6 h (*n* = 3 for each group; *P < 0.05, **P < 0.01, ***P < 0.001, and ****P < 0.0001). **E–J** showed that the results and analyses of western blot of IL-6, TNF-α, Mincle, and p-syk in the LPS-induced BV2 cell line. **E** Representative immunoblot showing the IL-6, TNF-α, Mincle, syk, p-syk, p-NFκB-p65, NFκB-p65, and GAPDH protein expression; (F-J) The quantitative densitometric ratio of **F** IL-6, **G** TNF-α, **H** Mincle, relative to GAPDH, **I** p-syk relative to syk and **J** p-NFκB-p65 relative to NFκB-p65 (*n* = 3 for each group; *P < 0.05, **P < 0.01, and ****P < 0.0001)

Figure 7 shows the qPCR results for Mincle and its related inflammation factors and western blot for Mincle and its related inflammation factors and p-syk protein expression in six cell groups, including BV2 control, BV2 + LPS, BV2 + LPS + Q, BV2 + LPS + M, BV2 + LPS + (M + Q), and its control. The mRNA expression of Mincle and its related inflammatory factors (IL-6, TNF-α, and IL-1β) were assayed at 6 h in each cell group. Measured values of inflammatory cytokines and Mincle in LPS-stimulated BV2 cells increased significantly compared with the normal BV2 cells but significantly decreased in three treatment groups (Fig. 7A-D). There was no significance between BV2 + LPS + Q and BV2 + LPS + M groups, and the conditioned medium of MSCs with IronQ reduced the Mincle, IL-6, and TNF-α mRNA expression more remarkably than MSCs conditioned medium and quercetin intervention to the LPS-mediated BV2 cells.

Moreover, we further investigated the IL-6, TNF-α, Mincle, and p-syk protein expression levels (Fig. 7E-J and Additional file 5) via western blot treated with the conditioned medium of MSCs and MSCs with IronQ and quercetin intervention in LPS-induced BV2 cell line at 24 h. Figure 7E-J and Additional file 5 represent the results and analyses of the western blot. Compared with the BV2 control group, the expression levels of Mincle and p-syk proteins significantly increased in the LPS-induced BV2 cell line. Three treatments all downregulated the protein expression levels of IL-6, TNF-α, Mincle, and p-syk (*P* < 0.0001). The effect of the conditioned medium of MSCs with IronQ decreased these protein expression levels more significantly than the conditioned medium of MSCs compared with quercetin intervention.

### Conditioned medium of MSCs with IronQ exerted the therapeutic effects by regulating the Mincle/syk signaling pathway in LPS-induced BV2 cells

**Fig. 8:**
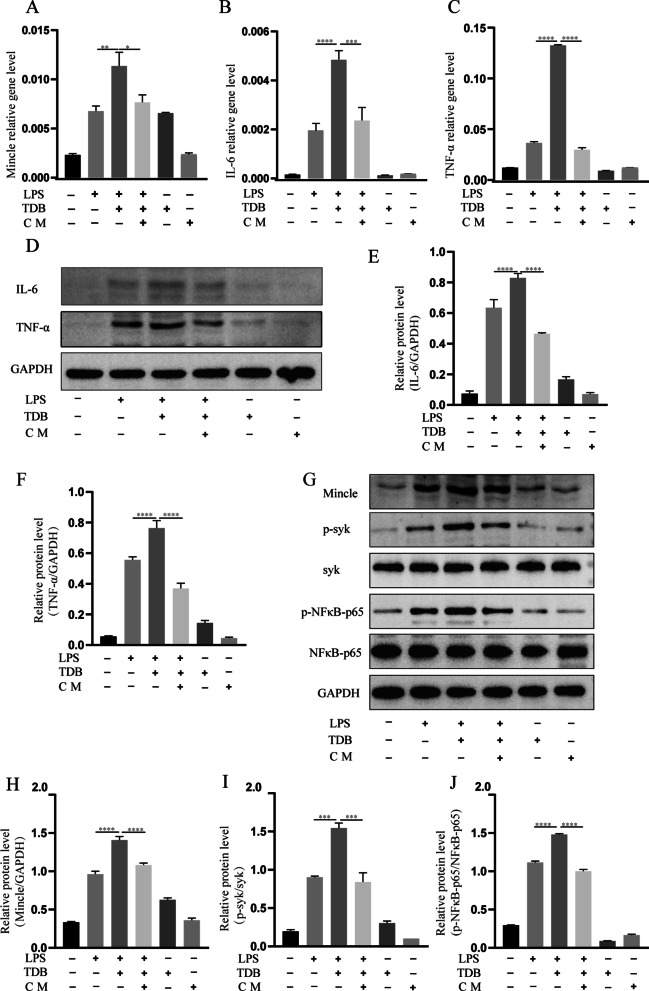
The results and analyses of qPCR and western blot revealed that the conditioned medium of MSCs with IronQ reduced inflammatory response by regulating the Mincle/syk signaling pathway. **A-C** Presentation of graphs of **A** Mincle, **B** IL-6, and **C** TNF-α mRNA expression levels in different cell groups after 6 h (*n* = 3 for each group; *P < 0.05, **P < 0.01, ***P < 0.001, and ****P < 0.0001). **D-J** The results and analyses of western blot demonstrated the protein expression levels of IL-6, TNF-α, Mincle, p-syk, and p-NFκB-p65 in each cell group. **D, G** Representative immunoblot showing the effect of conditional medium on protein expression of IL-6, TNF-α, Mincle, syk, p-syk, NFκB-p65, p-NFκB-p65, and GAPDH (**E–F, H–J**); the quantitative densitometric ratio of **E** IL-6, **F** TNF-α, **H** Mincle relative to GAPDH, **I** p-syk relative to syk, **J** p-NFκB-p65 relative to NFκB-p65 (*n* = 3 for each group; ****P < 0.0001). Conditional medium of MSCs with IronQ reduced Mincle and its downstream p-syk, and p-NFκB-p65 protein expression significantly compared with other treatment groups after 24 h

Figure 8 shows the results and analyses of qPCR and western blot for Mincle and its downstream in six cell groups. The mRNA expression levels of Mincle and its related inflammatory factors IL-6 and TNF-α were assayed at 6 h. The measured values of inflammatory factors and Mincle in inflammatory cell models significantly increased compared with the normal group. However, they decreased remarkably after administering the conditional medium of MSCs with IronQ (Fig. 8A-C). We further analyzed the protein expressions of IL-6, TNF-α, Mincle, syk, p-syk, NFκB-p65, and p-NFκB-p65 with western blotting after 24 h of LPS-induced BV2 cell line and MSCs + IronQ conditioned medium treatment. Figures 8D-J and Additional file 6 represent the results and analyses of the western blot in this experiment. Mincle and its downstream p-syk and p-NFκB-p65 were significantly upregulated in the LPS-induced BV2 cell line. The conditioned medium of MSCs with IronQ significantly induced a down-expression of Mincle, p-syk, and p-NFκB-p65 compared with other treatment groups (*P* < 0.0001).

## Discussion

Previously, many studies have verified that the molecular mechanisms of MSCs transplantation protect against brain damage displaying therapeutic effects. However, the challenges transplanted MSCs face with the harsh environment of injured tissue may further induce poor survival of the transplant cells and blur the successful therapy. We hypothesized that a combination of MSCs with bioactive chemical compounds could promote the therapeutic effects of MSCs transplantation in brain damage. Here, we utilized a novel theranostic agent named IronQ to combine (partly labeled) with the MSCs to inquire into the potential therapeutic mechanisms of the ICH-injured brain. IronQ, which consists of a molecule of iron (Fe3^+^) and quercetin, is a positive MRI contrast agent on T1. IronQ exhibits its excellent dual function as an MRI probe and a stimulating agent of PBMCs to differentiate proangiogenic cells [25, 26]. In this study, we found that the internalization into MSCs of IronQ at a specific concentration (200 μg/mL) can promote MSCs’ proliferation and be monitored via the T1-weighted MRI. Moreover, combined treatment of MSCs with IronQ attenuated the inflammation response to improve neurological deficits and brain edema in the ICH-induced perihematomal brain tissue by inhibiting the Mincle/syk signal axis. The results suggested that the magnetically labeled IronQ-MSCs are not only safe for cell tracking purposes. Nevertheless, MSCs combined with IronQ also play a synergistic role in improving neurological function.

Following ICH, perihematomal edema (PHE) happens rapidly, accompanied by a sharp growth of nearly 75% of its maximum volume in one day, peaks at three days, showing an absolute growth, and lasts up to 14 days [8, 41–44]. In humans, PHE is considered a radiological marker following ICH [45]. Therefore, the extent of PHE, both as a therapeutic target and a surrogate marker, is associated with poor outcomes after ICH [42, 46]. Thus, it is critical to decrease brain edema to protect against neurological deficits after ICH. Our research showed that the therapies, quercetin, MSCs, and MSCs + IronQ can alleviate the neurological deficits score and BWC in mice with ICH. The therapeutic efficacy of IronQ-labeled MSCs transplant and MSCs transplantation displayed a neuroprotective effect at the same level and significantly high efficacy than quercetin gavage treatment. In addition, the HE and Nissl staining also indicated that the neuroprotection of IronQ-labeled MSCs is superior to MSCs and quercetin treatment, respectively. Preclinical studies have reported that quercetin improves behavioral recovery by ameliorating inflammatory response after stroke [24, 47]. It has been demonstrated that MSCs improve neurological outcomes and modulate the immune cells, including microglia and neutrophils, to ameliorate inflammatory responses in rats after ICH [48, 49]. Therefore, according to the above analyses, our results suggest that IronQ could upregulate the neuroprotective effect displaying the synergistic effect of decreasing PHE.

Previous studies have reported the axonal injury and demyelination in an ICH rat model induced by collagenase via immunofluorescent staining, and the authors found that apparent axonal damage and demyelination occurred inside and around the hematoma within 3 days, in which there was substantial neuron death [50]. Moreover, Tao et al. have reported that the obvious demyelination and axonal damage on the third day in rats after primary brainstem hemorrhage (BSH) were extremely associated with brain edema and neurofunctional dysfunction resulting from a hematoma [51]. They found that on the third day after BSH, there was an evident reduction of MBP staining, which can be used to detect intact axonal myelin in the brain region around the hematoma [51]. It has been verified that reactive astrogliosis, which will be modulated to promote brain repair and reduces neurological impairment via the production of neurotrophic substances, is a pathological change of CNS injury [52].

Many reports have confirmed that MSCs transplantation can enhance the expression levels of NeuN [49], MBP [53], and GFAP [54] in brain injuries. Therefore, in order to know the neuroprotective effect of MSCs combined with IronQ after ICH, we further investigated the protein expression levels of MBP (the marker of myelin), NeuN (the marker of neurons), and GFAP (the marker of astrocytes) through the immunofluorescence and western blot, which are associated with recovery of neurological function [55]. The results showed that the protein expression of NeuN, MBP, and GFAP decreased remarkably in mice after ICH. However, an obvious expression increase of NeuN, MBP, and GFAP was seen after giving combined treatment compared with the MSCs transplantation and quercetin gavage. Therefore, this study indicated that the combined treatment might present a synergistic role for the improvement of the survival of neurons, repairation of myelin, and activation of astrocytes, suggesting that combined therapy may ameliorate the hemorrhagic-induced brain damage by promoting the survival of neurons, remyelination, and neurotrophic function of astrocytes. In the CNS, the most abundant astrocytes have multifaceted roles for providing nutrients, recycling neurotransmitters, and fulfilling homeostasis and have been regarded as increasingly important regulators of neuronal functions [52]. Our results are consistent with previous studies. Following the administration of MSCs with IronQ for mice with ICH, astroglial–mesenchymal phenotype switching of astrocytes enhanced the ability of proliferation to protect them from apoptosis [54, 56]. However, some studies have suggested that reactive astrocytes induce glial scar formation when excessive proliferation exists, which is harmful to neural network reconstruction and axon growth [57, 58]. It suggests that reactive astrocytes display a double-edged sword function.

Inflammation initially results in cell swelling, damage, and then further brain edema, as described in ICH [59]. Once the released blood components infiltrate into the parenchyma, an immediate inflammatory response is followed via mobilized and activated inflammatory cells [60]. Microglia, as the primary immune cell source of the innate immune system, participate in the pathological process of ICH and response to neuroinflammation [61, 62].

In the process of infection and tissue damage, one of the pattern-recognition receptors, named Mincle, is mainly expressed on the surface of microglia/macrophages and upregulated [32, 63–67]. It binds to endogenous antigens, recruiting and activating its downstream (syk), and then activates the NFκB pathway to activate innate immunity and host defense, which ultimately generates biologically active inflammatory factors for the induction of inflammatory responses [38, 68–70]. The results from other reports demonstrated that the Mincle/syk signaling pathway positively responds to brain damage-induced innate immune inflammation in many brain diseases, such as IS, TBI, and SH [30–33, 71]. Our study revealed that Mincle protein and its downstream proteins were significantly increased in mice with ICH. However, treatment of ICH mice with the MSCs transplantation or quercetin gavage decreased the expression levels of Mincle/Syk signaling pathway-related protein. Quercetin is generally known as an anti-inflammatory and anti-oxidative agent, possessing anti-inflammation and immunosuppressive effects on macrophage and peripheral blood mononuclear cells [72, 73]. Preconditioned MSCs with quercetin could augment the cold burn wound therapeutic effect of MSCs by reducing inflammation and enhancing neovascularization [74]. This result suggests the effects of quercetin on improving cell-based therapy. A previous study reported that quercetin inhibits the activity of the Mincle/syk signaling pathway, which further suppresses the production of inflammatory factors in macrophages [23]. Consistent with previous findings, our results showed that the combined treatment of MSCs with IronQ significantly restrained the Mincle/syk signaling. The combination treatment also decreased the related proteins associated with inflammation in mice brain tissue following hemorrhage compared with the MSCs transplantation and quercetin gavage. Our findings suggested that the combined treatment of MSCs with IronQ through the Mincle/Syk signaling pathway mitigates ICH-induced neuroinflammation and improves neural function. Consequently, the results from this study demonstrated that the combined treatment has synergistic effects on regulating Mincle/syk signaling pathway for improving brain damage in mice with ICH. Moreover, neurological defects and brain swelling were significantly moderated before blocking the Mincle signaling in CNS injury [31, 67], suggesting that Mincle signaling can be regarded as a promising therapeutic target during ICH-induced neuroinflammation.

Our research has a certain extent limitation. Foremost, the Mincle/syk signal axis investigation and its modulation were restricted to the mice with ICH. It is critical to further extend to other experimental animals to verify the function of Mincle/syk signaling. Next, we did not acquire additional evidence by constructing Mincle and syk viruses. Mincle/syk signal is an attractive target requiring further studies in the subsequent investigation of ICH.

In general, we provide evidence for the first time that the iron-quercetin complex name IronQ is safe for labeling mesenchymal stem cells and tracking the labeled cells in mice with ICH using MRI with T1-weighted techniques. In addition, the first report that combined treatment of MSCs transplantation with IronQ via IronQ-labeled MSCs playing a synergistic role improves ICH-induced brain injury, which is associated with suppression of the Mincle/syk signaling pathway in mice following ICH-induced brain injury. The results of this study consolidated the therapeutic effects of combined treatment of MSCs with IronQ and supplied positive insights for comprehending the potential molecular mechanisms and helping us to develop more specific targeted drugs. Moreover, the findings also give us more confidence in the administration and monitoring of the stem cells as precise at the target site via MRI in preclinical and clinical research and contribute to treatments for alleviating the neurological outcomes following ICH.

## Conclusions

This study provided a strategy to enhance the neuroprotective effects of MSCs in mice with ICH. It also demonstrated that the combined treatment plays a synergistic role in improving the ICH-induced inflammatory consequences through the downregulation of the Mincle/syk signaling pathway.

## Supplementary Information

**Additional file 1.** Original microscope figures (supported Fig. 2B-D) for MSCs and identified MSCs^IronQ^ via Prussian blue staining.

**Additional file 2.** Original western blot gels of Fig. 4D for protein expression levels of NeuN, MBP, and GFAP in different ICH mice brain tissue groups.

**Additional file 3.** Original western blot gels of Fig. 5E for protein expression levels of inflammatory factors (IL-6 and TNF-α) in different ICH mice brain tissue groups.

**Additional file 4.** Original western blot gels of Fig. 6B for protein expression levels of Mincle/syk signaling pathway via the transplantation of MSCs with IronQ for ICH mice model.

**Additional file 5.** Original western blot gels of Fig. 7E for protein expression levels of inflammatory factors, Mincle and its downstream in LPS‐induced BV2 cells through the intervention of conditioned medium of MSCs combined with IronQ.

**Additional file 6.** Original western blot gels of Fig. 8DG for the protein expression levels of Mincle/syk signaling pathway via the intervention of conditioned medium of MSCs with IronQ to LPS‐induced Mincle-overexpressed BV2 cells.

## Data Availability

All data generated or analyzed during this study are included in this published article [and its Additional files].

## Abbreviations

ICH: Intracerebral hemorrhage
MSCs: Mesenchymal stem cells
MRI: Magnetic resonance imaging
IronQ: Iron-quercetin complex
LPS: Lipopolysaccharide
BWC: Brain water content
TCM: Traditional Chinese Medicine
PBI: Primary brain injury
SBI: Secondary brain injury
CNS: Central nervous system
