# Supplementary figures and images for: Mesenchymal stem cells transplantation combined with IronQ attenuates ICH-induced inflammation response via Mincle/syk signaling pathway
Source: Stem Cell Res Ther. 2023 May 15;14:131. doi: 10.1186/s13287-023-03369-6 (PMC10186667; doi:10.1186/s13287-023-03369-6)

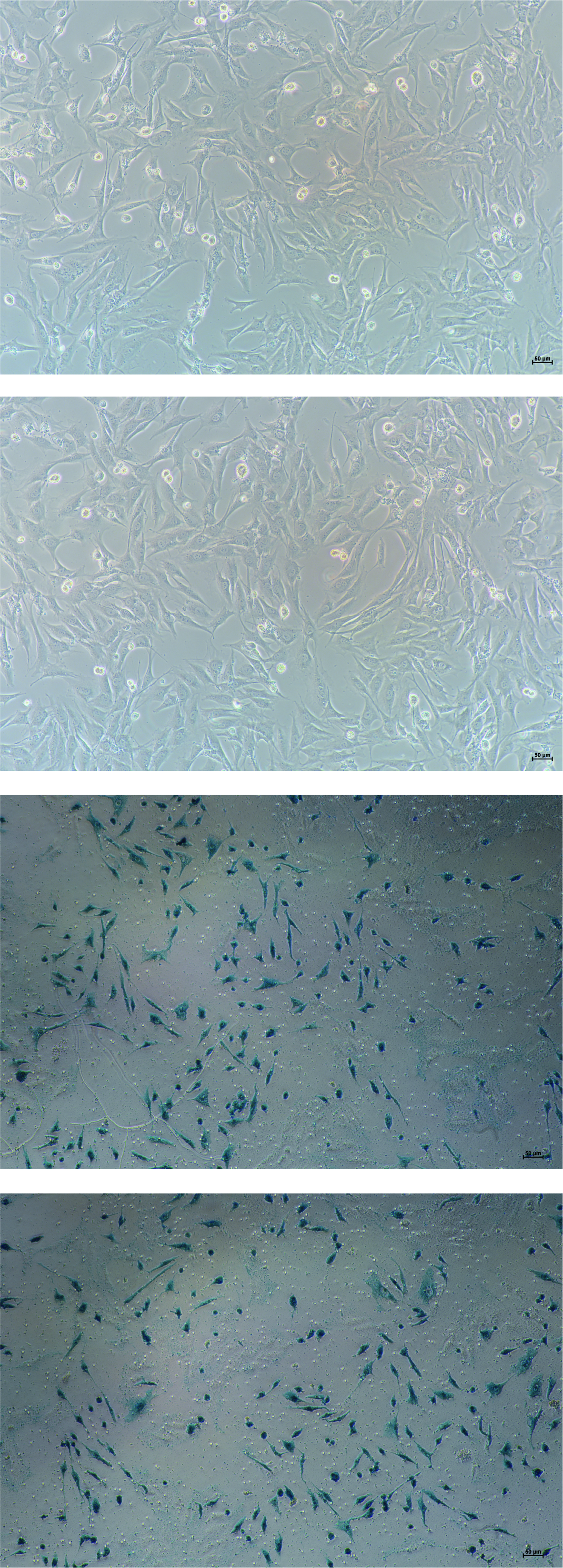

Supplement: Supplementary file 1 — Additional file 1. Original microscope figures (supported Fig. 2B-D) for MSCs and identified MSCsIronQ via Prussian blue staining. [file 13287_2023_3369_MOESM1_ESM.tif]

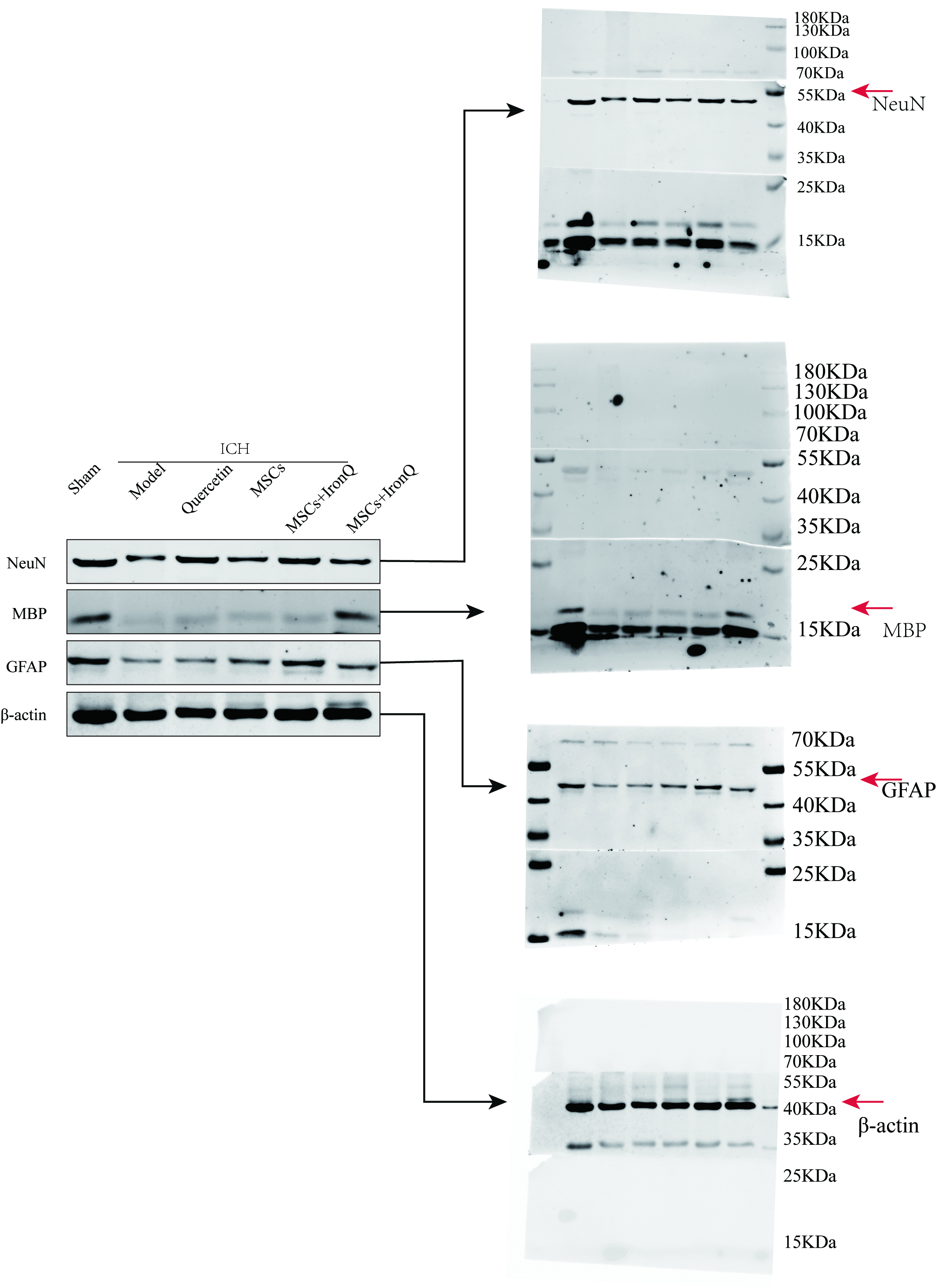

Supplement: Supplementary file 2 — Additional file 2. Original western blot gels of Fig. 4D for protein expression levels of NeuN, MBP, and GFAP in different ICH mice brain tissue groups. [file 13287_2023_3369_MOESM2_ESM.tif]

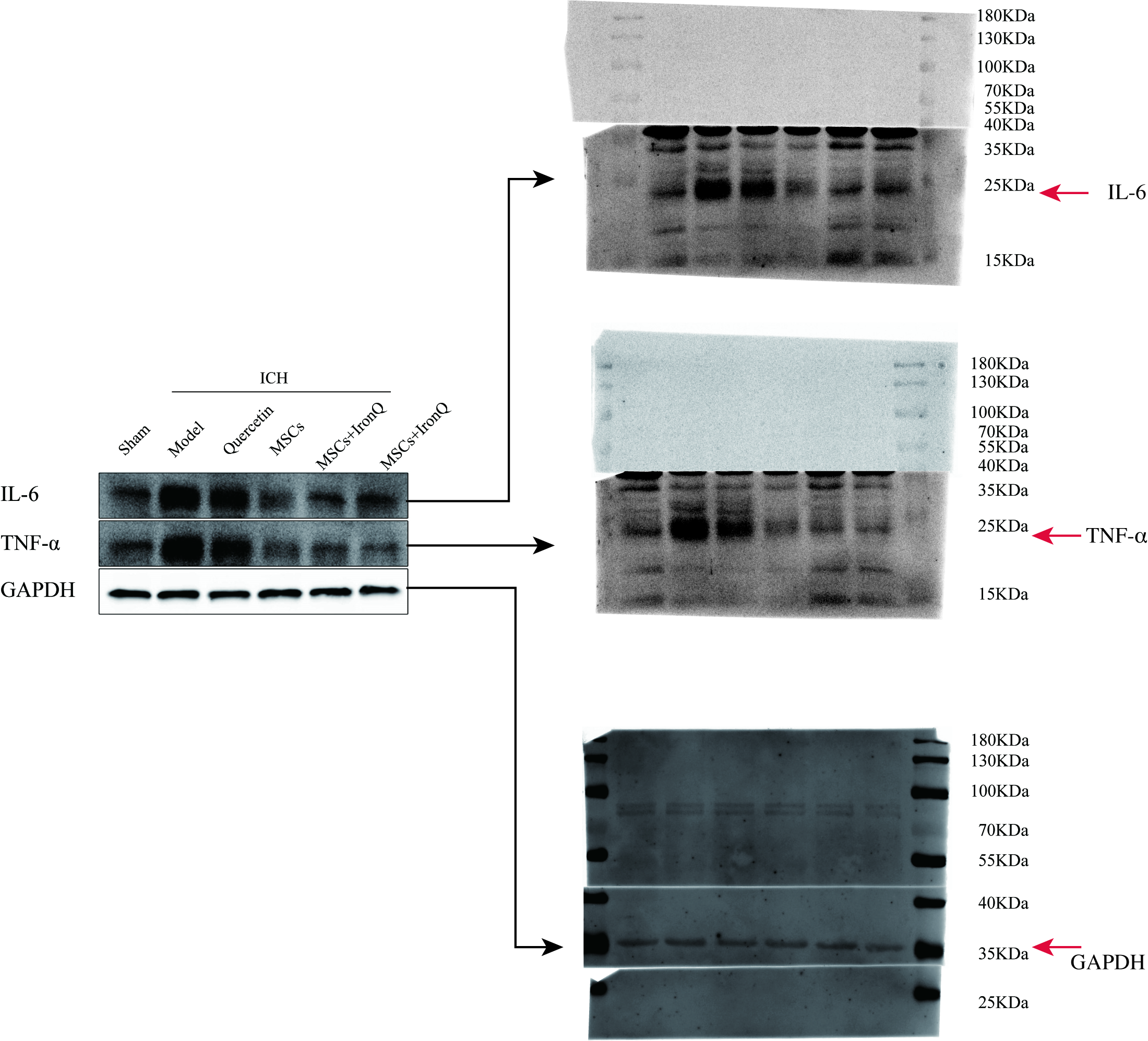

Supplement: Supplementary file 3 — Additional file 3. Original western blot gels of Fig. 5E for protein expression levels of inflammatory factors (IL-6 and TNF-α) in different ICH mice brain tissue groups. [file 13287_2023_3369_MOESM3_ESM.tif]

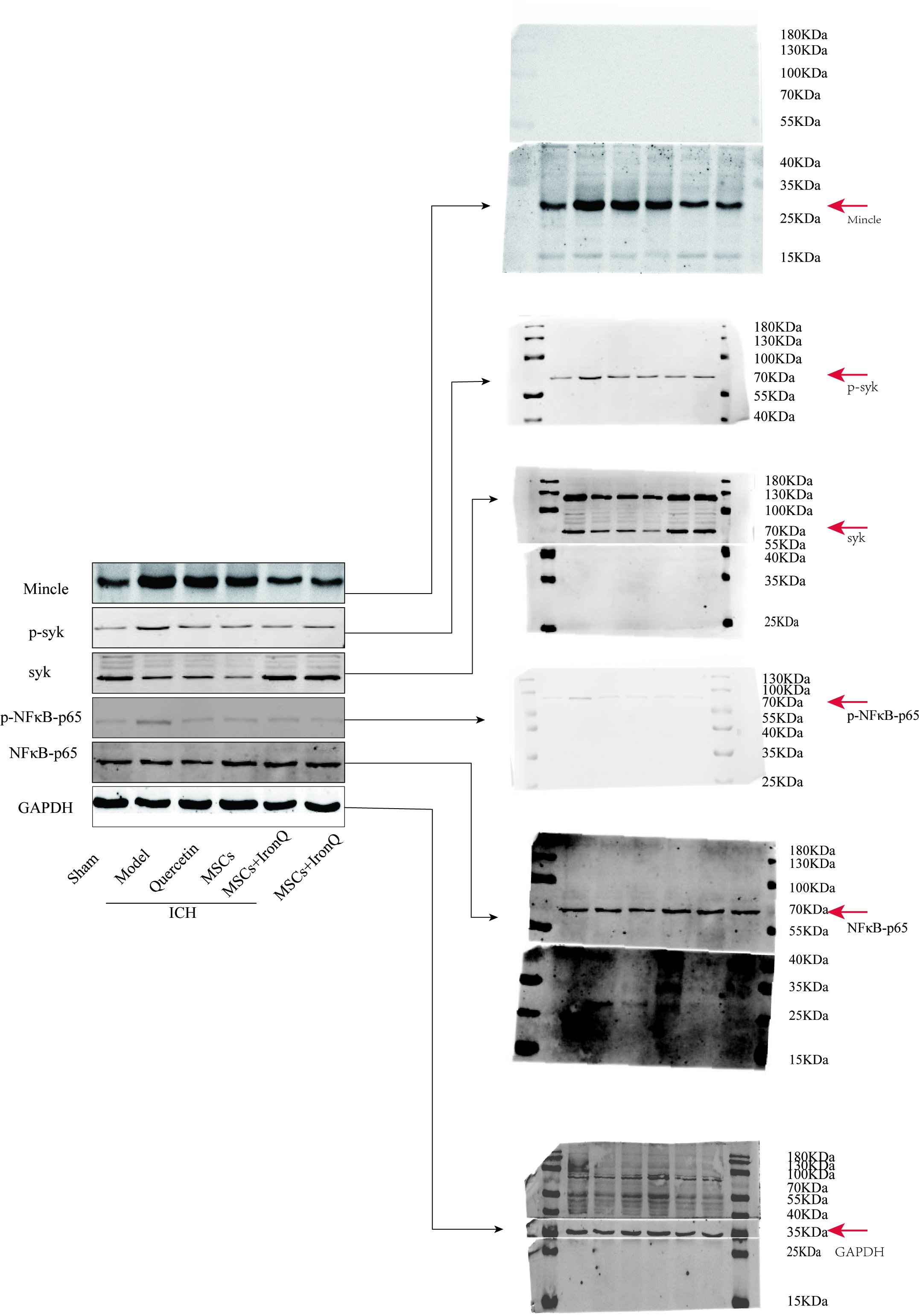

Supplement: Supplementary file 4 — Additional file 4. Original western blot gels of Fig. 6B for protein expression levels of Mincle/syk signaling pathway via the transplantation of MSCs with IronQ for ICH mice model. [file 13287_2023_3369_MOESM4_ESM.tif]

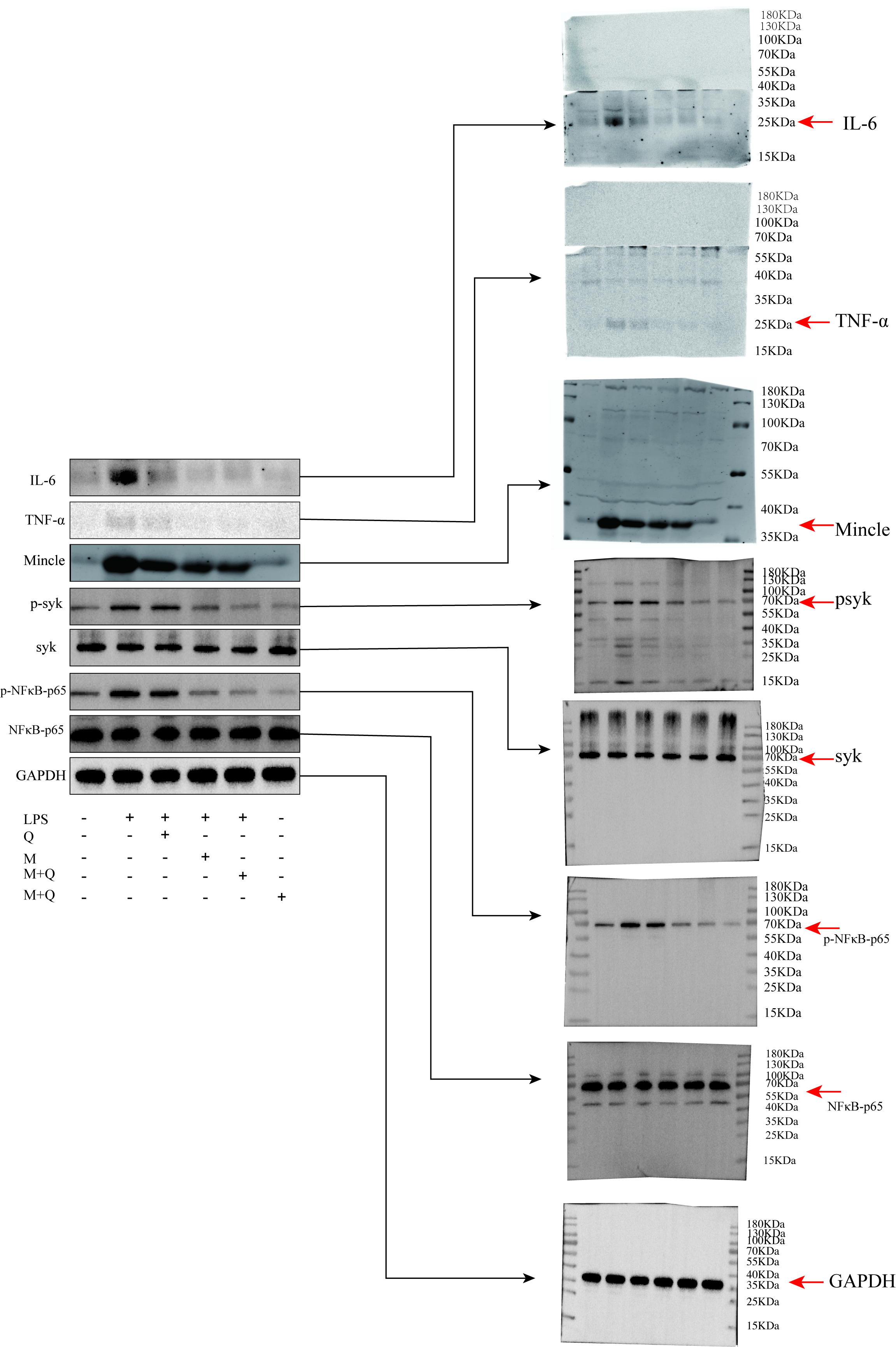

Supplement: Supplementary file 5 — Additional file 5. Original western blot gels of Fig. 7E for protein expression levels of inflammatory factors, Mincle and its downstream in LPS‐induced BV2 cells through the intervention of conditioned medium of MSCs combined with IronQ. [file 13287_2023_3369_MOESM5_ESM.tif]

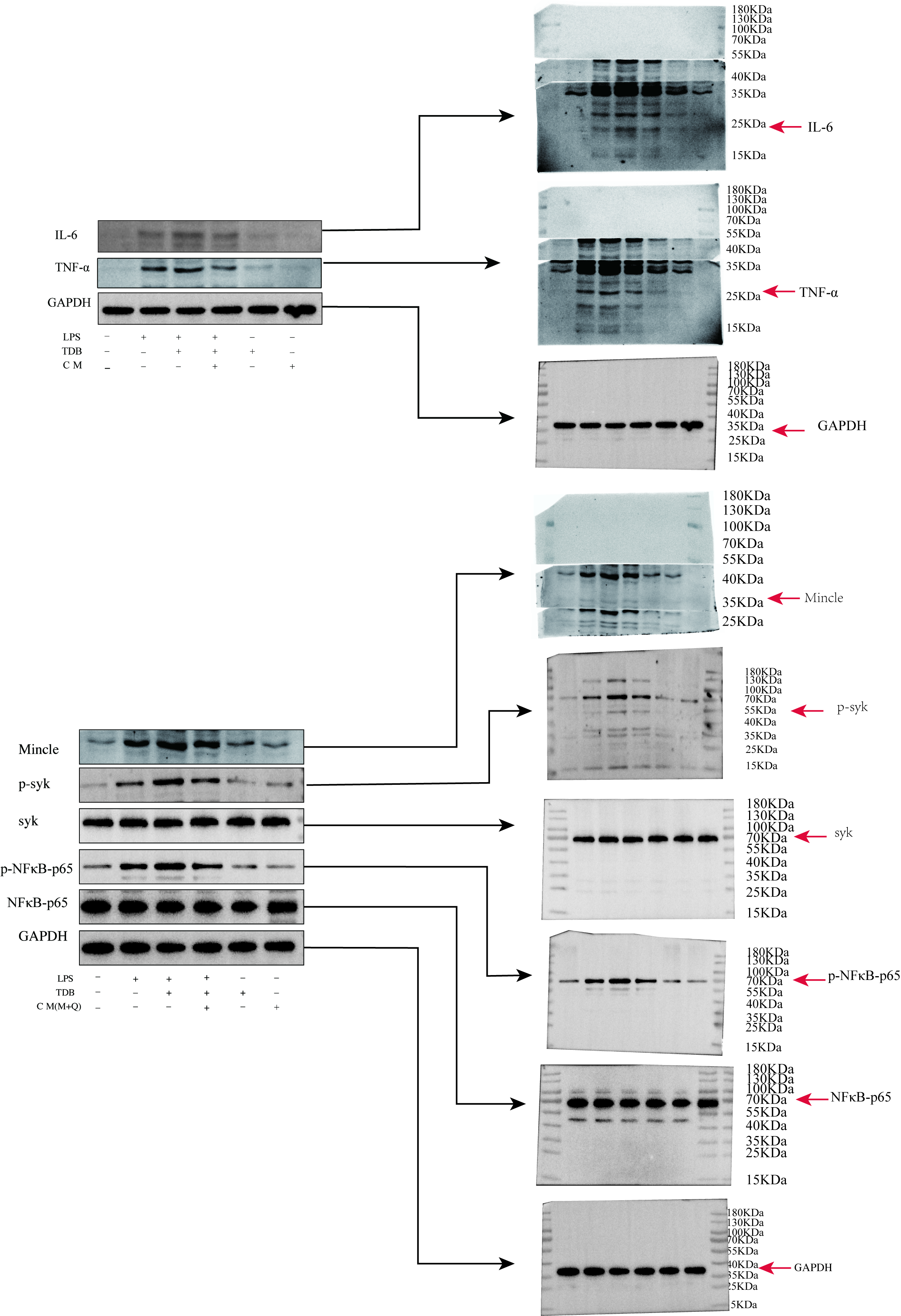

Supplement: Supplementary file 6 — Additional file 6. Original western blot gels of Fig. 8DG for the protein expression levels of Mincle/syk signaling pathway via the intervention of conditioned medium of MSCs with IronQ to LPS‐induced Mincle-overexpressed BV2 cells. [file 13287_2023_3369_MOESM6_ESM.tif]
